# Effect of Nitrogen, Phosphorous, and Light Colimitation on Amphidinol Production and Growth in the Marine Dinoflagellate Microalga *Amphidinium carterae*

**DOI:** 10.3390/toxins14090594

**Published:** 2022-08-28

**Authors:** Alejandro Molina-Miras, Alejandro Bueso-Sánchez, María del Carmen Cerón-García, Asterio Sánchez-Mirón, Antonio Contreras-Gómez, Francisco García-Camacho

**Affiliations:** 1 Department of Chemical Engineering, University of Almería, 04120 Almería, Spain; 2 Research Center CIAIMBITAL, University of Almería, 04120 Almería, Spain

**Keywords:** microalgae, dinoflagellate, *Amphidinium carterae*, growth model, amphidinols, raceway photobioreactor

## Abstract

The marine dinoflagellate microalga *Amphidinium carterae* is a source of amphidinols, a fascinating group of polyketide metabolites potentially useful in drug design. However, *Amphidinium carterae* grows slowly and produces these toxins in tiny amounts, representing a hurdle for large-scale production. Understanding dinoflagellate growth kinetics under different photobioreactor conditions is imperative for promoting the successful implementation of a full-scale integrated bioproduct production system. This study evaluates the feasibility of growing *Amphidinium carterae* under different ranges of nitrogen concentration (NO_3_^−^ = 882–2646 µM), phosphorus concentration (PO_3_^3−^ = 181–529 µM), and light intensity (Y_0_ = 286–573 µE m^−2^ s^−1^) to produce amphidinols. A mathematical colimitation kinetic model based on the “cell quota” concept is developed to predict both algal growth and nutrient drawdown, assuming that all three variables (nitrogen, phosphorous and light) can simultaneously colimit microalgal growth. The model was applied to the semicontinuous culture of the marine microalgae *Amphidinium carterae* in an indoor LED-lit raceway photobioreactor. The results show that both growth and amphidinol production strongly depend on nutrient concentrations and light intensity. Nonetheless, it was possible to increase *Amphidinium carterae* growth while simultaneously promoting the overproduction of amphidinols. The proposed model adequately describes *Amphidinium carterae* growth, nitrate and phosphate concentrations, and intracellular nitrogen and phosphorus storage, and has therefore the potential to be extended to other systems used in dinoflagellate cultivation and the production of bioproducts obtained therein.

## 1. Introduction

Marine dinoflagellates are primary producers that play a crucial role in oceanic phytoplankton communities. These microalgae have recently attracted increasing attention because they are candidates for biodiesel production [1], municipal wastewater and aquaculture effluent treatments [2], and above all, because they produce a wide variety of impressive bioactive compounds [3]. In particular, dinoflagellates of the genus *Amphidinium* are an important source of amphidinols, a unique group of polyketide metabolites that exhibit antifungal, anticancer, and hemolytic activity that can be used in biomedical, toxicological and pharmacological research [4]. 

Due to their many potential applications, the demand for such products has increased significantly in recent years. Nonetheless, mass production is extremely difficult and still lagging behind the practical requirements [5]. At the laboratory and pilot-scale, dinoflagellates are usually cultivated in different types of closed photobioreactors, typically bubble columns and flat panels with large surface-volume ratios, for the small-scale production of various dinoflagellate bioproducts [6,7,8,9]. However, to take advantage of the amphidinols and other potential bioactive substances, cost-effective industrial-scale culture systems that provide an appropriate environment for these highly shear-sensitive microalgae will have to be used [3,6].

Raceway reactors (of various sizes) have been the most widely used systems for the large-scale production of non-dinoflagellate microalgae destined for diverse applications. However, as far as we know, no attempts have been made to culture dinoflagellates in large raceway systems. While they are simple and easy to operate, improved performance is necessary to further optimize their operation and reduce their energy and nutrient consumption [10]. It has been reported that the main limitations for the large-scale production of microalgal biofuels are the huge amounts of nutrients and water required [11], while nutrient balance and nutritional supplementation are known to affect bioactive compound productivity [12]. Therefore, the main objective of culture system optimization should be to maximize production, although it is also crucially important to ensure healthy growth and metabolism. The nutritional requirements for healthy growth and bioproduct production greatly depend on the particular dinoflagellate species; hence, it is imperative to find the appropriate medium formulation. Nitrogen and phosphorus are essential nutrients that are critical to microalgal metabolic function and they take part in essential biochemical processes; thus, their concentrations in the culture are fundamental aspects that determine microalgal growth and biomass production [1]. Nitrogen is used to build nucleic acids, amino acids, and proteins, whereas phosphorus is an important constituent of phospholipids and nucleic acids [13] as well as being used for energy transport. It has been reported that both nitrogen and phosphorus can potentially limit [14] and colimit [15] phytoplankton growth in natural water environments. Nitrogen and phosphorus colimitation has also been observed in monospecific cultures [13,16].

The effects of multi-nutrient limitation on phytoplankton growth have been studied for many years. It is usually described using Liebig’s law of the minimum (also referred to as the threshold model), assuming that growth is always controlled by the most limiting resource; thus, it does not include the nutrient colimitation of growth. Many authors have followed this approach to model phytoplankton growth rates (see [13]). Multi-nutrient limitation can also be described by the multiplicative model, assuming that growth can be controlled by several nutrients simultaneously. This concept has also been widely used to develop kinetic models [17]. In autotrophic cultures, it is not only chemical resources but also light intensity that is essential for microalgal growth, and the balance between the chemical nutrients and the light intensity will strongly determine the overall metabolism of the microalgal cells. This has been recognized as an area of interest for further work and modeling [18].

Clearly, mathematical models that consider multiple factors, and that are capable of representing microalgal growth in terms of both nutrient and light limitation, provide a better explanation of growth than a model that only considers the effect of a single limiting factor. These models are vital in representing photobioreactor performance and in optimizing and scaling-up processes, helping to find the best operating conditions and identifying sensitive parameters [10]. While different models have been proposed to understand microalgae growth, there is a lack of consensus on how different variables should be combined to describe it; hence, it remains an important research area (see [17], and references therein). Indeed, there is a conspicuous need for mathematical models that help us understand and simulate microalgal growth kinetics as a function of the nitrogen and phosphate concentrations as well as the light intensity available. Consequently, the objective of this work is to investigate the simultaneous influence of nitrogen, phosphorous, and irradiance on growth, pigment content, and amphidinol production, and to develop a comprehensive deterministic colimitation kinetic model that describes the effect of multiple nutrients on transient microalgal growth. The model proposed here accurately predicts microalgal biomass growth and nutrient drawdown and can serve as a tool to rationally develop reproducible systems that have enhanced biomass productivities. The semicontinuous culture method used in this study clearly promoted an increase in growth along with high amphidinol production rates. The hemolytic activity of the obtained biomass correlated well with the amphidinol production. To estimate the model’s kinetic parameters, we used a database compiled from semicontinuous culture experiments on the marine dinoflagellate *A. carterae* in an indoor raceway photobioreactor [19]. An approach for balancing the model’s complexity and the database was also addressed.

## 2. Results and Discussion

### 2.1. Growth Kinetics and Modeling

**Figure 1:**
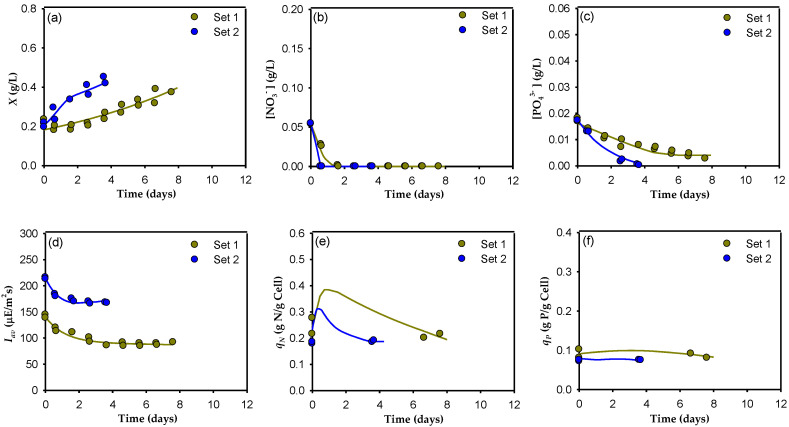
Dynamic profiles for Sets 1 and 2 of the semicontinuous culture of the microalga *A. carterae*. The data from two sequential cultures for each set (shown in Table 1) are superimposed. The temporal changes in the cell concentration (**a**), the dissolved nitrate (**b**) and dissolved phosphate concentrations (**c**) in the supernatant, the average irradiance available for the cells (**d**), the nitrogen cell quota (**e**), and the phosphate cell quota (**f**) are shown. Solid lines represent the predictions of the proposed model.

**Figure 2:**
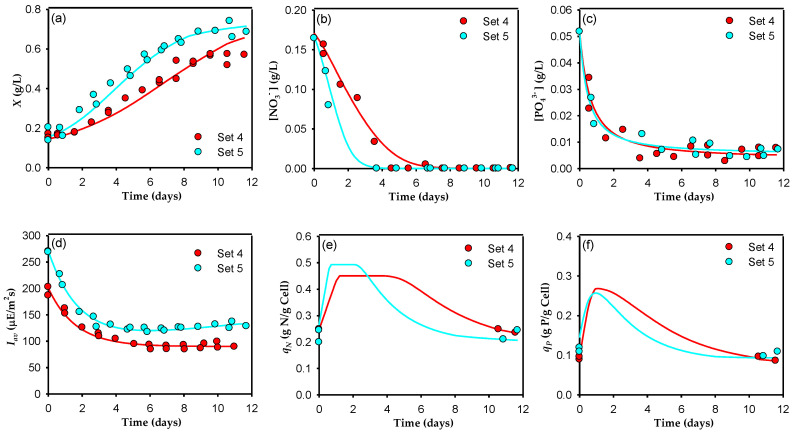
Dynamic profiles for sets 4 and 5 of the semicontinuous culture of the microalga *A. carterae*. The data from two sequential cultures for each set (shown in Table 1) are superimposed. Temporal changes in the cell concentration (**a**), the dissolved nitrate (**b**) and dissolved phosphate concentrations (**c**) in the supernatant, the average irradiance available for the cells (**d**), the nitrogen cell quota (**e**), and the phosphate cell quota (**f**) are shown. Solid lines represent the predictions of the proposed model.

**Figure 3:**
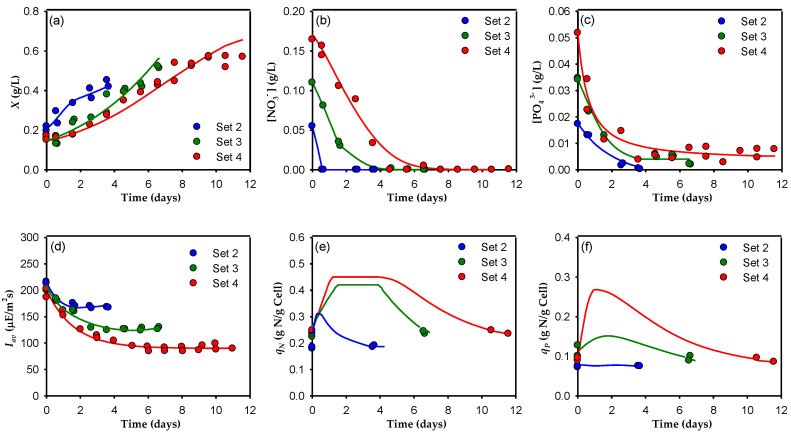
Dynamic profiles for sets 2, 3, and 4 of the semicontinuous culture of the microalga *A. carterae*. The data from two sequential cultures for each set (shown in Table 1) are superimposed. Temporal changes in the cell concentration (**a**), the dissolved nitrate (**b**) and dissolved phosphate concentrations (**c**) in the supernatant, the average irradiance available for the cells (**d**), the nitrogen cell quota (**e**), and the phosphate cell quota (**f**) are shown. Solid lines represent the predictions of the proposed model.

A summary of the experimental conditions used to develop the model is shown in Table 1. The experiments were grouped into five sets. Different set combinations can be selected, as shown in Figure 1, Figure 2 and Figure 3, according to the factors explored in each: (i) Sets 1 and 2 were used to study the effect of light intensity, *Y*_0_ (286 µE m^−2^ s^−1^ with a light/dark cycle of 12:12 and 430 µE m^−2^ s^−1^ with a light/dark cycle of 18:6) using a regular f/2 culture medium with an N:P = 5; (ii) sets 4 and 5 were used to evaluate the effect of *Y*_0_ (430 µE m^−2^ s^−1^ with a light/dark cycle of 18:6 and 573 µE m^−2^ s^−1^ with a light/dark cycle of 24:0) using f/2 × 3 as the culture medium with an N:P = 5; and (iii) sets 2, 3, and 4 were used to compare the effects of the nutrient concentration (regular f/2, f/2 × 2, and f/2 × 3) in the culture medium with an N:P = 5 and a constant value of *Y*_0_ (430 µE m^−2^ s^−1^ with a light/dark cycle of 18:6). In natural systems, microalgae are exposed to dynamic environments, and they can only be successfully grown if they can adapt their physiological processes and growth kinetic characteristics to the environmental fluctuations [20]. As shown in the previous study [19], the transitions between the different modes of operation and between the different sets of semicontinuous experiments induced adaptation-acclimation phenomena in the cells due to changes in the medium composition and the irradiance conditions. Since the model developed here is designed for quasi-steady-state responses, to minimize the influence of cell adaptation-acclimation when changing the culture conditions between the different sets of experiments, only the last two cultures of each experimental set were used to develop the model. To clearly compare the different sets, the cultures in this work are presented in Figure 1, Figure 2 and Figure 3 as independent batch cultures and assumed to start at time zero. As far as we know, this study is the first that attempts to use a multiplicative model to describe dinoflagellate growth and nutrient exhaustion by considering the effect of nitrogen, phosphate, and available light intensity colimitation; consequently, the kinetic parameters for this system are not available in the literature. Moreover, the growth kinetics of a microalga, as with the growth kinetics of microbial cells, cannot be represented by a single set of kinetic parameters, independently of the cell history or current environmental conditions, as is frequently done (see [21]). Therefore, in this work we estimated the model parameters using Equations (5)–(13) and the in-house data [19], obtaining a group of parameter values for each data set. The results obtained are shown in Table 2.

Figure 1 presents a comparison of Sets 1 and 2. Figure 1a shows the significant influence of available irradiance, *I_av_* (depicted in Figure 1d) on the biomass concentration, *X*, when using regular f/2 medium. As expected for a limiting nutrient, an increase in *I_av_* significantly improves microalgal growth. Clearly, *I_av_* kinetic limitation is observed, with *µ_max_* = 0.1 d^−1^ in Set 1 and *µ_max_* = 0.495 d^−1^ in Set 2 (a huge increase of almost 500%) showing that *A. carterae* effectively grows at high light intensities, unlike other dinoflagellates that grow better under low light intensities (<40–50 µE m^−2^ s^−1^) [22,23].

**Figure 4:**
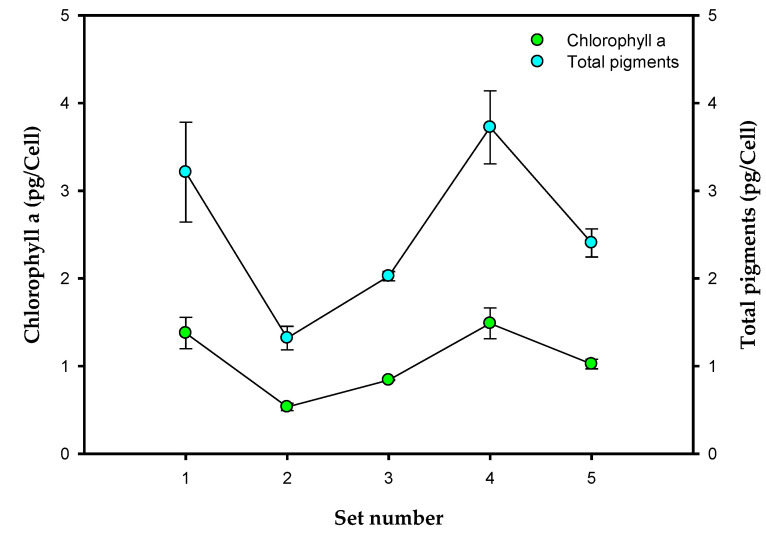
Effect of the culture medium composition and the irradiance level on chlorophyll *a* and total pigments (in terms of specific contents). Data points are averages, and vertical bars are the standard deviation for duplicate samples.

Cultures of both sets were also stoichiometrically limited by nitrogen and phosphorus, reaching similar *X_max_* values when the nitrate and phosphate were exhausted, at 0.39 and 0.44 g L^−1^, respectively (a difference of “only” 13%), but at different culture times. Interestingly, the nitrate in the culture medium was exhausted during the first few hours of cultivation in both sets (Figure 1b) but this occurred more rapidly in cultures that had a higher *I_av_* (Set 2) (Figure 1d), with maximum uptake rates of 0.305 and 0.409 gNO_3_^−^ gcell^−1^ d^−1^ for sets 1 and 2, respectively. Evidently, irradiance had a positive effect on nitrate assimilation for cell synthesis. Furthermore, in both cases, the cells continued to grow substantially after the nutrient in the medium was completely depleted. This growth, which is uncoupled from nutrient uptake, is a well-known capability of microalgae, including dinoflagellates, due to their luxury uptake of nutrients [23,24,25]. The cells were able to store nitrogen internally, as shown in Figure 1e, where one can observe that the model predicts an increase in *q_N_* concomitant with the withdrawal of nitrates from the medium, reaching a higher value in those cultures that grew more slowly (Set 1), but apparently without saturating storage capacity in any case. When the nitrates were completely removed from the medium, the cells began to metabolize the nitrogen stored within them. When growth stopped, a residual *q_N_* of around 0.2 gN gcell^−1^ was observed in both sets. It is important to note that, in set 2, a relevant decrease in pigments, including chlorophyll *a*, was observed compared to set 1, as shown in Figure 4. This pigment decrease, and concomitant *I_av_* increment when the *Y_0_* increased, revealed a photoacclimation pattern that agreed well with previous findings [26].

Regarding phosphate, total depletion was also observed at the end of the set 1 cultures (Figure 1c) with a maximum consumption rate of 0.021 gPO_4_^3−^ gcell^−1^ d^−1^. However, in the set 2 cultures, although a maximum consumption rate of 0.077 gPO_4_^3−^ gcell^−1^ d^−1^ was reached, the phosphate was not completely exhausted. As reported by [27], this indicates a nitrate-limited condition. As soon as the nitrate was depleted from the medium, the phosphate uptake was severely affected. In addition, although microalgae have been reported to store phosphates in the cells as polyphosphate granules [28], the model does not predict luxury uptake for phosphate, and the *q_P_* remained unchanged throughout the culture (Figure 1f). This clearly indicates that P was being metabolized as it was ingested by the cell, giving priority to the synthesis of cellular constituents [20] in the cultures of both sets; thus, *q_P_* remained constant throughout the cultures at a residual value of approximately 0.1 gP gcell^−1^. As mentioned before, both the nitrogen and the phosphorus uptake rates increased when irradiance intensity and the light-dark cycle increased, demonstrating a reduced capacity for uptake of both nutrients in the dark phase of the culture. These results are in line with those of [29] for the dinoflagellate *Prorocentrum micans*.

Figure 2 compares sets 4 and 5, which were carried out with higher irradiance and a nutrient concentration three times greater than those of Sets 1 and 2. The behavior of the cells in Sets 4 and 5 was parallel to that of Sets 1 and 2, but with some important differences. The presence of a kinetic limitation in cell growth due to irradiance (Figure 2a) was also observed, since the Set 4 cultures had lower *I_av_* than those of Set 5 (Figure 2d), and the *µ_max_* in Set 4 was 0.198 d^−1^ compared to a *µ_max_* of 0.306 d^−1^ in the Set 5 cultures (an increase of “only” 55%), along with a more important stoichiometric limitation than between Sets 1 and 2—in Set 4, an *X_max_* of 0.56 g L^−1^ was reached whereas, in Set 5, it was approximately 0.72 g L^−1^ (an increase of 25%). Because the initial nitrate concentration was higher than in Sets 1 and 2, the nitrate from the medium took longer to deplete (Figure 2b), although the nitrate uptake consumption rate was seen to increase along with increasing *I_av_*, from 0.441 to 0.618 gNO_3_^−^ gcell^−1^ d^−1^ for Sets 4 and 5, respectively. Like the cultures in Sets 1 and 2, the cultures in Sets 4 and 5 continued to grow after the nitrates were depleted, pointing to the presence of luxury uptake. As the nitrate disappears from the medium, the model predicts an increase in the cell quota (Figure 2e) with storage capacity saturation apparently being dependent on the *I_av_* (0.473 gN gcell^−1^ in Set 4 and 0.484 gN gcell^−1^ in Set 5).

The phosphate concentration decreased in a similar way in the cultures of both sets (Figure 2c) to a residual value of around 0.004 g L^−1^ soon after the nitrate in the culture medium was exhausted. In these cultures, the model does predict an increase in *q_P_* (Figure 2f), again similar in both sets, but apparently without reaching the storage limit for this nutrient. Like Sets 2 and 1, a decrease in pigments was observed in Set 5 relative to Set 4, including chlorophyll *a* (Figure 4). However, this decrease was lower than in Set 2 relative to Set 1. Similar results were reported by [30], with these authors showing that in continuous cultures, the pigment content of *Dunaliella tertiolecta* was less affected by an increase in light intensity when there was a high nitrogen concentration.

Figure 3 shows a comparison of Sets 2, 3, and 4 when carried out at an equal illumination intensity and dark/light cycle, and with an increasing concentration of nutrients. When the *X* profile is compared, a clear kinetic limitation is observed (Figure 3a) directly related to the *I_av_* in the cultures (Figure 3d), in accordance with previous finding [31,32], with *µ_max_* = 0.495, 0.391, and 0.198 d^−1^ in Sets 2, 3, and 4, respectively. The decrease in *I_av_* with increasing initial N and P concentrations is concomitant with the increase in chlorophyll *a*, and the total pigments (Figure 4). This positive relationship between pigments and N and P has been reported by many authors for dinoflagellates [33] as well as for other microalgae (see [30]). A stoichiometric limitation related to the availability of nutrients was also observed, with an *X_max_* of 0.43, 0.52, and 0.58 g L^−1^ in Sets 2, 3, and 4, respectively. In this case, one could also clearly see that the cultures continued to grow after the nitrates were exhausted from the culture medium due to the presence of luxury uptake. As the initial concentration of nitrates increased, the nitrate in the medium took longer to deplete (Figure 3b) although the maximum uptake rates were similar in the three sets: 0.409, 0.390, and 0.441 gNO_3_^−^ gcell^−1^ d^−1^ in Sets 2, 3, and 4, respectively. As the nitrate disappears from the medium, the model predicts an increase in *q_N_* (Figure 3e), reaching storage capacity saturation in Sets 3 and 4, with similar maximum cell quota values for both (0.42 gN gcell^−1^ in Set 3 and 0.44 gN gcell^−1^ in Set 4). The phosphate concentration decreased with the increase in *X* (Figure 3c), but in this case, the consumption rates were clearly higher in the cultures with higher phosphorus concentrations, having maximum uptake rates of 0.077, 0.205, and 0.327 gPO_4_^3−^ gcell^−1^ d^−1^, respectively. It was also observed that there was a decrease in the phosphate concentration down to a residual value of around 0.004 g L^−1^ soon after the nitrate in the culture medium was exhausted. The luxury uptake of P made *q_P_* increase more in the cultures that had a higher initial P concentration in the medium (Figure 3f). However, there was apparently no storage capacity saturation for phosphate.

The model simulation results obtained using the optimized parameters for the concentrations of biomass, nitrates, phosphates, *I_av_*, and nitrogen and phosphorus cell quotas are represented by solid lines in Figure 1, Figure 2 and Figure 3. As these figures show, the observed consistency of the model simulations with the experimental data demonstrates the model’s ability to adequately reproduce general trends under a variety of irradiance and nutrient conditions. One can see that the model reproduces algal growth responses to different nitrate and phosphate concentrations, as well as to different irradiance cycles and levels (Figure 1a, Figure 2a and Figure 3a, respectively). In all cases, there is a short lag phase roughly equivalent to the time needed to fill the internal nitrogen and phosphate quotas, following an exponential growth phase (as in a true batch culture) with higher *µ_max_* for higher *I_av_*; this is in good agreement with previous studies carried out on other microalgae [34] that showed an increase in the final biomass concentration at the highest nitrate and phosphate concentrations. The model also adequately fits nitrate and phosphate drawdown (Figure 1b,c, Figure 2b,c and Figure 3b,c). It reproduces the greater affinity of *A. carterae* to uptake nitrate rather than phosphate. Clearly, it was the nitrate that was first exhausted from the culture medium, increasing its cell quota. This behavior is probably due to the low N:P ratio used (5), far below 16, the threshold below which nitrogen is considered limiting [35]. Figure 1e,f, Figure 2e,f and Figure 3e,f show the results obtained for the *q_N_* and *q_P_*. In these figures, although only two experimental points are available for each experiment (the initial quota and the final quota), the simulated dynamics are typical, replicating the ability of microalga to store nitrogen and phosphorus when they are abundant, similar to the results reported by other authors [36]. Except for the *q_P_* in Sets 1 and 2, the cell quota shows a pronounced increase at the beginning of the cultures due to the rapid uptake of nitrogen and phosphate during the lag growth phase. During the exponential growth phase, *q_N_* remained approximately constant, and later decreased when the nitrogen was depleted from the culture medium. The fact that both quotas generally decrease in parallel shows that both nutrients limit growth simultaneously. This behavior indicates that there is a complex relationship between *µ*, *X_max_*, *I_av_*, *q_N_*, and *q_P_*, which supports the use of a multiplicative-type model such as that represented by Equation (5). 

**Figure 5:**
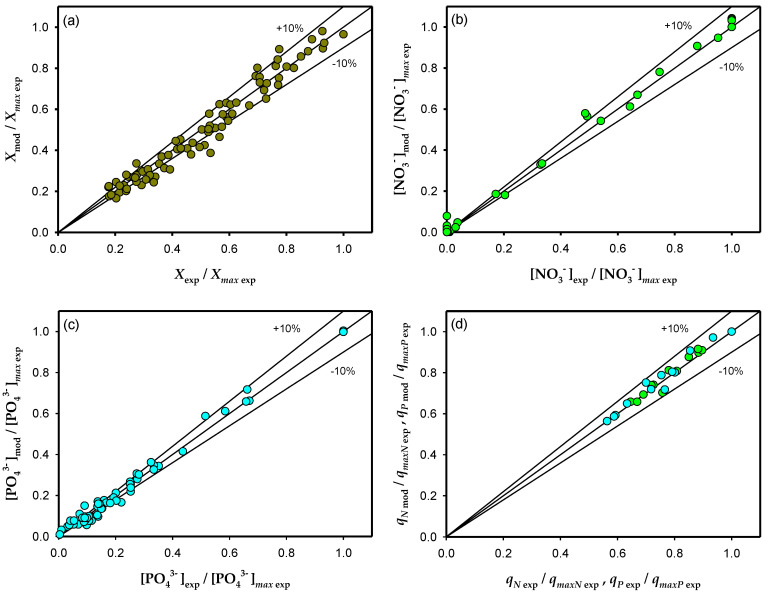
The model prediction versus the experimental normalized cell concentration (**a**), the dissolved nitrate concentration in the supernatant (**b**), the phosphate concentrations in the supernatant (**c**), and the nitrogen and phosphate cell quota (**d**).

As one can observe in Figure 5, the microalgal growth, the consumption of nutrients (nitrates and phosphates), and the dynamics of the internal nitrogen and phosphorus quotas are very well represented by the model proposed in this work, correlating around 90% of the data with an error of less than 10%. The model’s capability to describe *A. carterae* growth, the time course of the nutrient concentrations, and the cell quotas when the environmental conditions change, suggests that the basic features of microalgae physiology are appropriately represented.

### 2.2. Amphidinol Production

**Figure 6:**
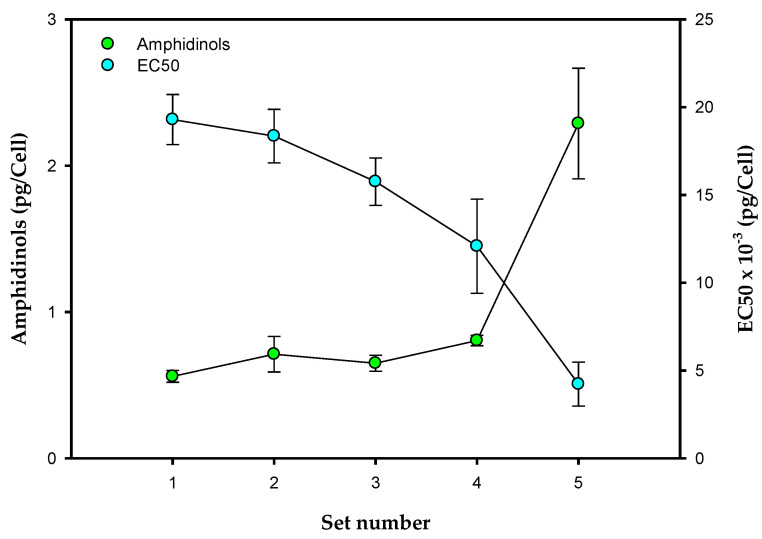
Effect of the culture medium composition and the irradiance level on amphidinols, and the EC50 (i.e., the number of *A. carterae* cells per well giving 50% hemolysis). Data points are averages and vertical bars are the standard deviation for duplicate samples.

Amphidinols are secondary metabolites. Their production is not necessary for cell growth and they are usually synthetized during the late growth phase. It is accepted that they play a key role in the interaction of cells with their environment and appear to serve as a chemical defense against competitors and predators. The cellular content of amphidinols is shown in Figure 6. One can observe that minimal amphidinol accumulation was obtained in Set 1, where the lowest irradiance, nitrate, and phosphate concentrations were used. The increased daily irradiance in Set 2 produced a slight increase in amphidinol production, parallel to the decrease in pigments due to photoacclimation. The increased nutrient availability in Sets 3 and 4 had no significant impact on the accumulation of amphidinols, but further increased irradiance in Set 5 showed an almost four-fold increase in the amphidinol content, clearly due to the photoacclimation observed, as discussed above. Although many compounds could be potentially responsible for the hemolytic activity, it did closely correlate with the amphidinol content. As shown in Figure 6, EC50 decreased concomitantly with the increasing amphidinol content from Set 1 to Set 5. Maximum hemolytic activity was reached in Set 5 (with an EC50 value of 4 × 10^3^ cell well^−1^); this is about one order of magnitude higher than that reported by [9] for a different strain of *A. carterae*. The observed lack of contribution to hemolytic activity from other compounds in these cultures has been previously confirmed using supervised multivariable data analysis techniques, obtaining a linear correlation between the amphidinol titers in methanol:water (80:80 *v*/*v*) extracts and hemolytic activity data [4]. The cell growth dynamic shown in Figure 1, Figure 2 and Figure 3, and the amphidinol production dynamic shown in Figure 6 describe a culture pattern strongly controlled by the availability of nitrogen, phosphorus and irradiance, consistent with previous finding regarding the stoichiometry regulation of phytoplankton toxins [32,37]. This culture methodology clearly promotes the biosynthesis of amphidinols, suggesting that overproduction of amphidinols is compatible with cell growth, as recently shown in fed-batch cultures of *A. carterae* [38].

It is well recognized that despite the efforts made over recent years, dinoflagellate cultures are still difficult to manage due to the intrinsic characteristics that differentiate them from microorganisms. These include quite low rates of growth and low biomass productivities, an intricate metabolism resulting in low bioproduct production, and the propensity to store nutrients uncoupled from growth (which makes it difficult to use classic models such as the Monod model). Furthermore, when they grow under light/dark cycles, they synchronize cell division and divide at the same time at the end of the dark period, then grow during the light period (which affects the elemental increase in biomass); they also adapt their pigment classes and contents, and their secondary metabolites, to fluctuations in irradiance [39]. Clearly, dinoflagellate growth is far from balanced during the life of the culture and the dinoflagellates have unique metabolic requirements that are difficult to provide for; they also complicate photobioreactor performance forecasting in the absence of a growth model.

## 3. Conclusions

This work demonstrates that manipulating the main operating conditions of nutrient formulation and light intensity can increase growth and promote amphidinol production in *A. carterae*, while providing relevant information that is potentially useful in the current efforts to improve dinoflagellate culture performance in photobioreactors, a necessary step to establishing suitable sources of amphidinols and other bioproducts. A kinetic model was developed based on a comprehensive mathematical formulation to predict *A. carterae* growth dynamics under complex semicontinuous operation in a raceway photobioreactor. This instrument can help us to understand the intricate, synergistic interactions between nutrients and light intensity, and their combined influence on *A. carterae* growth. A bioprocess strategy such as this may also be extended to other dinoflagellates that produce different amphidinols or other bioproducts, serving as a general framework that can be used for effective optimization and process control, as well as for photobioreactor design and description. This will allow us to accurately calibrate investment in industrial-scale facilities where these microalgae are cultivated.

## 4. Materials and Methods

### 4.1. Database

A pre-existing database was used in this work. It was created from culture experiments carried out in an LED-based PVC paddlewheel-driven raceway photobioreactor to optimize the semicontinuous culture conditions of *A. carterae*. The database comprised experiments that were conducted over 170 days using different light/dark cycle combinations and different initial nitrate and phosphate concentrations in the cultures. Briefly, the marine microalga *A. carterae* Dn241EHU, which produces amphidinol A and amphidinol B [40] was obtained from the microalgae culture collection of the Department of Plant Biology and Ecology at the University of the Basque Country, and subsequently used in this study. Inocula were maintained in regular f/2 medium with an N:P ratio of 24, as previously described [19]. The raceway photobioreactor had a growth surface of 0.44 m^2^ and a culture volume of 33 L. The culture depth was 7.5 cm to alleviate light attenuation. Multicolor LED strips located outside the reactor were used to illuminate the culture, providing the desired light intensity. The pH was controlled at 8.5 by the on-demand addition of CO_2_ from behind the paddlewheel. The temperature was kept at 21 ± 1 °C using thermostatic water circulating through a steel tubular loop located inside the photobioreactor. Further details of the system are provided by [19].

The semicontinuous culture mode covered the period from day 27 until the end of the cultivation (day 172). Semicontinuous cultures were carried out removing a variable volume of culture and replenishing it with fresh medium to obtain an initial biomass concentration of 10^6^ cell mL^−1^ in all the experimental sets. This protocol was repeated each time the culture reached the stationary phase. To prepare the fresh medium, the nitrate and phosphate concentrations in the consumed medium were measured and regular f/2 medium was added with stock solutions of nitrates and phosphates to reach the desired concentrations. Similarly, the other nutrients were assumed to be totally consumed so the necessary amounts were added to reach the concentrations of the selected formulation. The experimental conditions for this work are summarized in Table 1. Further details can be found in [19].

### 4.2. Model Variables

The experimental values of the model variables were obtained from [19]. Briefly, the cell culture and biomass concentrations (*X*, cells mL^−1^ or g L^−1^) were quantified by flow cytometry and gravimetric dry-weight estimation, respectively. The phosphate and nitrate in the supernatants were determined according to protocols 4500-P and 4500-N, respectively, proposed by the American Public Health Association (APHA, 1995). The nitrogen (N) and phosphorus (P) content percentages in the biomass were determined by NOCHSP analysis and a modified chemical wet-oxidation method (CWO), respectively. Only experimental N and P data from the beginning and end of each semicontinuous culture were available. In the study presented here, the N and P percentages in the biomass allowed us to determine the cell quotas of *q*_*N*0_ and *q*_*P*0_ in Equations (12) and (13), respectively. *q*_*N*0_ and *q*_*P*0_ were expressed per unit of cell mass to avoid variability due to cell division synchrony under the different light/dark conditions [30]. Values for the average irradiance inside the culture (*I_av_*(*t*), µE m^−2^ s^−1^) were calculated from local irradiance measurements by applying a two-dimensional diffuse incident light model and assuming light attenuation inside the culture as described by the Beer–Lambert law for homogeneous media, whereas the mean daily irradiance (*Y*_0_) received by the culture was calculated as described by [19].

### 4.3. Analytical Measurements

The chlorophyll *a* and total pigments in the cells were determined as described in [19] using an HPLC apparatus equipped with a diode array detector. Amphidinols A and B were quantified from methanolic extracts of the harvested biomass through RMN analysis, as previously described [4], using a Bruker Avance III HD 600 spectrometer. The hemolytic activity of the cell extract was quantified using the erythrocyte lysis assay, as described elsewhere [9], with erythrocytes from defibrinated sheep blood. The values of the EC50 (i.e., the number of *A. carterae* cells per well giving 50% hemolysis) and the saponin control were calculated. The EC50 obtained for saponin control was 8.5 × 10^6^ ± 0.6 × 10^6^ pg per well. An equivalent saponin potency (expressed as pg saponin per *A. carterae* cell) can be calculated by dividing the EC50 for saponin by the EC50 for *A. carterae*.

### 4.4. Growth Model

It is generally accepted that increasing a model’s complexity does not necessarily enhance its efficiency. This is particularly true when the model is developed ad hoc from a pre-existing database, as was the case here. The explanation should be no more complex than that needed to explain the phenomenon; that is to say, the model should not be more complex than that supported by the data. Traditionally, kinetic models have assumed that a single factor controls the rate of microalgal growth. As a result, many kinetic models representing microalgal growth were developed as a function of a single variable, mainly the concentration of the limiting nutrient. The Monod model [41], initially intended for steady-state growth, is a general kinetic model that has been traditionally used to describe the relationship between the microalgal growth rate and the concentration of the limiting nutrient in the culture media. Since the nutrient concentration in the culture media is easily measured, the Monod model is widely applied, offering numerical stability and straightforward parameterization. However, the Monod model cannot simulate growth inhibition at high nutrient concentrations or represent growth in a way that is uncoupled from nutrient uptake, a well-known phenomenon in microalgae. These limitations have prompted the development of models that relate cell growth to the nutrient availability in the cell’s interior using the “cell quota”—the amount of a nutrient present in a cell. One advantage of these models is that they are applicable when the nutrient transfer rate into the cell is equal to the consumption rate, and when, in the absence of an external nutrient, growth continues due to the accumulated nutrient inside the cell. The Droop model is representative of cell quota models and has been used successfully in many different environments to model algae growth as a function of the intracellular concentration of nutrients (see [17]). The Droop equation [42] can be written as:

(1) $$\mu =\mu_{max,\;D}\left(1-\frac{q_{min}}{q}\right)$$

where *µ* is the growth rate, *q* is the current quota, *q_min_* is the minimum quota, and *µ_max,D_* is the theoretical maximum growth rate for the Droop model at the infinite quota; assuming that when *q* = *q_max_* and *µ* = *µ_max_*, the Droop equation can be normalized [43] to remove *µ_max,D_*, and thus Equation (1) can be written as:

(2) $$\mu =\mu_{max}\frac{\left(1-\frac{q_{min}}{q}\right)}{\left(1-\frac{q_{min}}{q_{max}}\right)}$$

The Caperon–Meyer model [44] is another quota model, essentially derived from the Michaelis–Menten enzyme kinetic equation, which can be written as:

(3) $$\mu =\mu_{max,CM}\frac{\left(q-q_{min}\right)}{K_{Q}+\left(q-q_{min}\right)}$$

where *µ_max,CM_* is the theoretical maximum growth rate for the Caperon–Meyer model, conceptually similar to *µ_max,D_* in the Droop model, and *K_Q_* is an additional curve-defining parameter with the same units as *q*. According to Flynn [45], Equation (3) can also be normalized in a similar way to Equation (2) to eliminate *µ_max,CM_*, and can thus be written as:

(4) $$\mu =\mu_{max}\frac{\left(1+K_{q}\right)\left(q-q_{min}\right)}{\left(q-q_{min}\right)+K_{q}\left(q_{max}-q_{min}\right)}$$

where *K_q_* is a dimensionless constant, unlike *K_Q_* in Equation (3). As shown by [43], the form of the curve represented by Equation (2) is increasingly hyperbolic as the difference between *q_max_* and *q_min_* increases. However, Equation (4) is more flexible and can represent any curve, from linear to rectangular hyperbolic, irrespective of the *q_max_* and *q_min_* values [43].

The different quota models have been extensively used and have been shown to satisfactorily reproduce microalgae growth in a constant environment [39]. However, these models fail to represent the growth of microalgae in dense cultures when photolimitation is present, and the effect of irradiance must be included in the model. Traditionally, to describe nitrogen–phosphorus colimitation on microalgae growth, the threshold theory has usually been adopted, whereas to explain nitrogen-light co-limitation, the multiplicative theory has often been used [35]. Therefore, in this work, to accurately describe algal growth, the commonly observed colimitation of nitrogen, phosphorus, and light in natural environments is assumed, using a multiplicative model, supposing that all three resources can simultaneously influence the overall growth rate. The interaction between the limitation effects of nitrogen and phosphorus on the cell quota and the saturation effects of light intensity are taken into account in the specific growth rate expression, as shown below:

(5) $$\;\;\;\mu =\mu_{max}\left(\frac{\left(1+K_{qN}\right)\left(q_{N}-q_{minN}\right)}{\left(q_{N}-q_{minN}\right)+K_{qN}\left(q_{maxN}-q_{minN}\right)}\right)\;\left(\frac{\left(1+K_{qP}\right)\left(q_{P}-q_{minP}\right)}{\left(q_{P}-q_{minP}\right)+K_{qP}\left(q_{maxP}-q_{minP}\right)}\right)\left(\frac{I_{av}^{n}}{K_{I}^{n}+I_{av}^{n}}\right)$$

where the subscript *N* represents nitrogen, and the subscript *P* refers to phosphorus; *I_av_* is the average irradiance inside the culture; *n* is a form parameter and *K_I_* is the half saturation constant for irradiance. The *I_av_* influence is described according to [46]. Interestingly, the formulation of Equation (5) implies that no growth takes place at night and, consequently, nutrients can be taken up during this period without being consumed for growth, leading to an increase in the cell quota. Accordingly, the net algal biomass growth rate can be expressed as:

(6) $$r_{X\;}\left(t\right)=\mu \left(t\right)X\left(t\right)$$

Since semicontinuous cultures are susceptible to nitrogen and/or phosphorus limitation or deficiency, an approach similar to that suggested by [47] was followed, in which it is also assumed that the nutrient uptake rates depend on extracellular and intracellular nutrient concentrations. Michaelis–Menten functional forms are used to calculate both the nitrogen and phosphorus uptake rates as follows:

(7) $$r_{N}\left(t\right)=r_{maxN}\frac{\left[{\mathrm{NO}}_{3}^{-}\right]\left(t\right)\frac{q_{N}\left(t\right)}{q_{maxN}}}{K_{pN}+\left[{\mathrm{NO}}_{3}^{-}\right]\left(t\right)\frac{q_{N}\left(t\right)}{q_{maxN}}}$$

(8) $$r_{P}\left(t\right)=r_{maxP}\frac{{\left(\left[{\mathrm{PO}}_{4}^{3-}\right]\left(t\right)\right)}^{n1}\frac{q_{P}\left(t\right)}{q_{maxP}}}{K_{pP}^{n1}+{\left(\left[{\mathrm{PO}}_{4}^{3-}\right]\left(t\right)\right)}^{n1}\frac{q_{P}\left(t\right)}{q_{maxP}}}$$

where *K_pN_* and *K_pP_* are the saturation constants for nitrate and phosphate, *n*_1_ is a form parameter, and $\left[{\mathrm{NO}}_{3}^{-}\right]\left(t\right)$ and $\left[{\mathrm{PO}}_{4}^{3-}\right]\left(t\right)$ are the nitrate and phosphate concentrations in the supernatant at a certain culture time, *t*. 

Semicontinuous cultures are described as successive batch cultures. Incorporating rate equations into the mass balance, the overall model proposed in this work is represented by a set of five ordinary differential equations describing the dynamics of microalgal biomass growth and nutrient consumption, as shown below:

(9) $$\frac{dX\left(t\right)}{dt}=r_{X}\left(t\right);\;\;\;\;\;\;\;\;\;\;\;\;\;\;\;\;\;\;\;\;\;\;\;\;\;\;\;\;\;\;\;\;\;\;\;\;\;\;\;\;\;\;X\left(0\right)=X_{0}$$

(10) $$\frac{d\left[{\mathrm{NO}}_{3}^{-}\right]\left(t\right)}{dt}=-r_{N}\left(t\right)X\left(t\right);\;\;\;\;\;\;\;\;\;\;\;\;\;\;\;\;\;\;\;\;\left[{\mathrm{NO}}_{3}^{-}\right]\left(0\right)={\left[{\mathrm{NO}}_{3}^{-}\right]}_{0}\;\;$$

(11) $$\frac{d\left[{\mathrm{PO}}_{4}^{3-}\right]\left(t\right)}{dt}=-r_{p}\left(t\right)X\left(t\right);\;\;\;\;\;\;\;\;\;\;\;\;\;\;\;\;\;\;\;\;\left[{\mathrm{PO}}_{4}^{3-}\right]\left(0\right)={\left[{\mathrm{PO}}_{4}^{3-}\right]}_{0}\;\;\;\;\;\;\;\;$$

(12) $$\frac{dq_{N}\left(t\right)}{dt}=-r_{N}\left(t\right)-\mu \left(t\right)q_{N}\left(t\right);\;\;\;\;\;\;\;\;\;\;\;\;\;q_{N}\left(0\right)=q_{N}{}_{0}\;$$

(13) $$\frac{dq_{P}\left(t\right)}{dt}=-r_{P}\left(t\right)-\mu \left(t\right)q_{P}\left(t\right);\;\;\;\;\;\;\;\;\;\;\;\;\;\;q_{P}\left(0\right)=q_{P}{}_{0}$$

where *X*_0_, ${\left[{\mathrm{NO}}_{3}^{-}\right]}_{0}$, and ${\left[{\mathrm{PO}}_{4}^{3-}\right]}_{0}$ are the initial biomass concentrations of dissolved nitrate and phosphate for each semicontinuous culture. The values of $q_{N}{}_{0}$ and $q_{P}{}_{0}$ represent the cell quotas of nitrogen and phosphorus, respectively, at the start of a semicontinuous culture. In summary, the set of equations from (5) to (13) represent the cell-quota-based growth model. The model parameters are *q_maxN_*, *q_minN_*, *q_maxP_*, *q_minP_*, *K_qN_*, *K_qP_*, *K_I_*, *K_pN_*, *K_pP_*, *µ_max_*, *r_maxN_*, *r_maxP_*, *n,* and *n*_1_. 

### 4.5. Database Treatment

As mentioned above, the model presented here consisted of a set of five ordinary differential equations (see Equations (9)–(13)) that involved four rate equations (from (5) to (8)). To meaningfully interpret the results from microalgal cultures, it is imperative to precisely estimate the rates. Unlike steady-state continuous culture systems, describing the kinetics of batch, fed-batch or semicontinuous cultures is much more demanding. Although our data sets contained structured and periodic measurements, certain measurements were missing at a few time points. Unavailable data need to be treated correctly, rather than just deleting any case that has missing values. Furthermore, the number of experimental points in each set, although sufficient for a rigorous (but qualitative) interpretation of the kinetics [19], might not be sufficient to fit the model with fourteen parameters. 

In order to overcome the above constraints, the regression imputation technique was employed. In regression imputation, the existing variables are used to make a prediction by means of simple equations. This approach preserves all the points by replacing the missing data with probable values estimated on the one side, allowing us to increase the amount of data available for modelling purposes on the other. The advantages of this approach are that no novel information is added, while the sample size can be increased, and the standard error reduced [48]. Taking into account the nature of the experimental data and their varying trend over time, different empirical equations have been used to relate them. Thus, algal growth, which includes the lag, log, deceleration, stationary, and death phases, is described by the following logistic equation [49]:

(14) $$X\left(t\right)=\frac{a_{1}}{1+exp\left(\frac{t-b_{1}}{c_{1}}\right)}$$

where *X*(*t*) is the biomass concentration at any time, *t* is time and *a*_1_, *b*_1_, and *c*_1_ are constants. Two equations were used to describe the time course of the nitrate concentration in the supernatant versus the culture time:

(15) $$\left[NO_{3}^{-}\right]\left(t\right)=a_{2}+b_{2}t$$

where *a*_2_ and *b*_2_ are constants, and

(16) $$\left[NO_{3}^{-}\right]\left(t\right)=a_{3}+b_{3}exp\left(-c_{3}t\right)+d_{3}t$$

where *a*_3_, *b*_3_, *c*_3_, and *d*_3_ are constants. Similarly, the following two equations were used to adjust the time course of the phosphate concentration data in the supernatant:

(17) $$\left[PO_{4}^{3-}\right]\left(t\right)=a_{4}+b_{4}t$$

where *a*_4_ and *b*_4_ are constants, and

(18) $$\left[PO_{4}^{3-}\right]\left(t\right)=a_{5}exp\left(\frac{b_{5}}{1+c_{5}}\right)$$

where *a*_5_, *b*_5_, and *c*_5_ are constants. Finally, the data on the average irradiance time course was fitted to the following equation:

(19) $$I_{av}\left(t\right)=a_{6}+b_{6}exp\left(-c_{6}t\right)+d_{6}t$$

where *a*_6_, *b*_6_, *c*_6_, and *d*_6_ are constants. Obviously, all the parameters in Equations (14)–(19) have no biological meaning. There was excellent agreement between the primary experimental data and the values derived from the above equations for all the datasets (*r*^2^ > 0.9). Thus, the database was rebuilt with the 257 points generated by this approach based on the regression imputation technique [48]. As mentioned in Section 4.2, the pre-existing database only allowed us to determine the nitrogen and phosphorus cell quotas at the start of each semicontinuous culture. Therefore, the only calibration data used for Equations (12) and (13) were *q*_*N*0_ and *q*_*P*0_.

### 4.6. Statistical Analysis

To rebuild the pre-existing database, Equations (14)–(19) (the regression imputation technique) were fitted to the corresponding experimental values using Statgraphics Centurion XVI software (StatPoint, Herndon, VA, USA). The rebuilt database used Berkeley Madonna^®^ v10 software (Berkeley Madonna Inc., Albany, CA, USA) to find the model parameter values (Equations (5)–(13)). The parameters were identified by minimizing the error between the experimental and the calculated data.

## Figures and Tables

**Table 1:** Experimental conditions of the 5 semicontinuous culture sets used in the present study. The “interval” column represents the time period for the repeated batch cultures. An incident light intensity, *I_0max_,* of 900 µE m^−2^ s^−1^ was used in all the experiments. The initial medium on day 29 was f/2 × 1; [PO_4_^3−^]_f/2_×5 (N:P = 5). At day 77, the medium was changed to f/2 × 2; [PO_4_^3−^]_f/2_ × 10 (N:P = 5). At day 99, the medium was changed again to f/2 × 3; [PO_4_^3−^]_f/2_ × 14.6 (N:P = 5).

Set	Interval (Days)	Culture Time (Days)	L/D Cycle (Hours)	*Y*_0_ (µE m^−2^ s^−1^)	[NO_3_^−^]_0_ (µM)	[PO_4_^3−^]_0_ (µM)
1	29		12:12	286	882	181
	43–51	0–8				
	51–58	0–7				
2	58		18:6	430	882	181
	69–73	0–4				
	73–77	0–4				
3	77		18:6	430	1764	362
	85–92	0–7				
	92–99	0–7				
4	99		18:6	430	2646	529
	99–110	0–11				
	110–122	0–12				
5	127		24:0	573	2646	529
	149–161	0–12				
	161–172	0–12				

**Table 2:** Summary of the kinetic parameter values obtained for the semicontinuous cultivation of *A. carterae*.

Parameter	Set 1		Set 2		Set 3		Set 4		Set 5	
*µ_max_* (d^−1^)	0.100	±0.004	0.495	±0.194	0.391	±0.012	0.198	±0.050	0.306	±0.036
*n* (-)	0.235	±0.022	0.080	±0.020	0.013	±0.006	0.465	±0.385	1.005	±0.867
*n*_1_ (-)	1.234	±0.086	1.271	±0.633	2.064	±1.156	4.175	±0.176	3.237	±1.114
*K_I_* (µE s^−1^ m^−2^)	7.79 × 10^−6^	±3.21 × 10^−6^	1.71 × 10^−4^	±1.11 × 10^−4^	1.65 × 10^−5^	±2.50 × 10^−6^	1.13 × 10^−4^	±5.99 × 10^−5^	1.19 × 10^−4^	±4.84 × 10^−5^
*K_qN_* (-)	0.013	±0.006	0.201	±0.042	0.070	±0.021	1.017	±0.331	1.138	±0.508
*K_qP_* (-)	2.58 × 10^−6^	±1.23 × 10^−6^	1.02 × 10^−2^	±5.03 × 10^−3^	2.87 × 10^−4^	±1.17 × 10^−4^	4.14 × 10^−5^	±1.65 × 10^−5^	6.09 × 10^−6^	±2.23 × 10^−6^
*q_minN_* (gN gcell^−1^)	0.005	±0.004	0.011	±0.008	0.152	±0.063	0.115	±0.018	0.194	±0.006
*q_maxN_* (gN gcell^−1^)	0.367	±0.015	0.312	±0.04	0.420	±0.013	0.473	±0.023	0.484	±0.012
*q_minP_* (gP gcell^−1^)	0.065	±0.046	0.072	±0.038	0.038	±0.034	0.118	±0.082	0.021	±0.011
*q_maxP_* (gP gcell^−1^)	0.132	±0.030	0.124	±0.016	0.240	±0.033	0.329	±0.003	0.402	±0.183
*r_maxN_* (gNO_3_^−^ gcell^−1^ d^−1^)	0.305	±0.097	0.409	±0.005	0.390	±0.071	0.441	±0.057	0.618	±0.276
*r_maxP_* (gPO_4_^3−^ gcell^−1^ d^−1^)	0.021	±0.009	0.077	±0.031	0.205	±0.054	0.327	±0.034	0.514	±0.066
*K_pN_* (gNO_3_^−^ L^−1^)	0.006	±0.004	0.006	±0.005	0.020	±0.015	0.093	±0.011	0.04	±0.029
*k_pP_* (gPO_4_^3−^ L^−1^)	0.007	±0.002	0.010	±0.003	0.057	±0.040	0.022	±0.004	0.045	±0.024
